# Analysis of Factors Influencing the Modulus of Hot-Recycled Asphalt Mixture with High RAP

**DOI:** 10.3390/ma16155280

**Published:** 2023-07-27

**Authors:** Zining Chen, Boying Liu, Decheng Feng, Gang Li

**Affiliations:** 1 China Road and Bridge Corporation, Beijing 100011, China; 2 China Communications Construction Company, Beijing 100120, China; 3 School of Transportation Science and Engineering, Harbin Institute of Technology, Harbin 150090, China

**Keywords:** hot-recycled asphalt mixture, regenerant, modulus, factor analysis

## Abstract

Generally, the dynamic modulus and bending stiffness modulus are used to evaluate the mechanical properties of asphalt mixture, and they are also used as basic parameters for asphalt mixture design. Therefore, a study was conducted on changes in the dynamic modulus and bending stiffness modulus of hot-recycled asphalt mixture with high levels of reclaimed asphalt pavement (RAP) under the influence of different factors: dosage of regenerant, curing temperature, and curing time. The performance of reclaimed asphalt pavement (RAP) was first evaluated. Then, the hot-recycled asphalt mixture was adjusted and designed in order to conduct modulus experiments, composed of the dynamic modulus test and three-point bending test. Finally, the influencing factors were not only qualitatively but also quantitatively analyzed to clarify the change laws of the mechanical parameters of hot-recycled asphalt mixture. The results showed that the modulus of the recycled asphalt mixture first decreased, then increased, and then decreased with increasing dosage of regenerant. As the curing time or temperature increased, the modulus first decreased and then increased. In terms of the dynamic modulus of the hot-recycled asphalt mixture, the curing time had the greatest impact, followed by dosage of the regenerant and curing temperature. For bending stiffness modulus, the influence of dosage of the regenerant was the greatest, followed by curing time and curing temperature. For the bending stiffness modulus of hot-recycled asphalt mixture, the curing conditions had a greater influence compared with the dynamic modulus.

## 1. Introduction

Asphalt pavement is widely used in high-grade highways due to its significant advantages such as comfortable driving, easy maintenance, and good smoothness. However, under cyclic vehicle loads and long-term exposure to harsh natural environments, asphalt pavement deteriorates and develops distress such as rutting, cracks, and potholes. During the maintenance and repair of asphalt pavement, large quantities of reclaimed asphalt pavement are produced. RAP has great recycling value because it contains non-renewable resources such as aged asphalt and aggregates. Its application in recycled asphalt mixture (especially hot-recycled asphalt mixture) has obvious economic and social benefits, and the performance of asphalt mixture is guaranteed [1,2,3,4]. Research has shown that when the RAP content in asphalt mixture is high, it can significantly reduce road construction costs by as much as approximately $94,000 per mile [5]. According to a report from the National Asphalt Pavement Association (NAPA) in the United States, approximately 89.2 million tons of RAP were used for recycled asphalt pavement in 2019, which saved over USD 3.3 billion [6]. Research found that with the increase in RAP content in recycled mixture, greenhouse gas emissions during the construction of asphalt pavement gradually decreased. When the RAP content was 50%, greenhouse gas emissions decreased by 20% [7]. A study by Al Qadi Imad L et al. reported that the performance of recycled asphalt mixture with RAP content less than 25% reached or even surpassed that of ordinary asphalt mixture [8].

When the proportion of RAP in the recycled mixture is high, it is necessary to add a regenerant to reactivate aged asphalt and restore its performance [9]. The degree of fusion of old asphalt after the addition of regenerant affects the modulus of recycled asphalt [10], and it is also necessary to determine the moduli of hot-recycled asphalt mixture, especially the dynamic modulus and bending stiffness modulus, which are key parameters in asphalt mixture design [11]. To determine the degree of fusion between aged asphalt and regenerant, the solvent extraction method was employed to obtain reactivated asphalt from RAP [12] and the mechanical properties of the reactivated asphalt were analyzed [13,14]. After studying the performance of recycled asphalt, Whiteoak D et al. found that the mixing of the regenerant and asphalt was influenced by three factors: mechanical mixing, diffusion, and compatibility between new and old aggregates; the extent of diffusion was the most critical factor [15,16]. Navaro et al. found that the temperature and the ratio of ultraviolet rays could affect the degree of fusion between aged asphalt and regenerant [17]. McDaniel et al. and Mogawer et al. assumed that the regenerant was completely mixed with aged asphalt, and they estimated the dynamic modulus of the mixture from the volume ratio of regenerant to aged asphalt to determine the dosage of RAP to add [18,19]. Furthermore, scholars have explored the aging mechanism of asphalt and the diffusion behavior of regenerant at the molecular scale, revealing the interaction mechanism between aged asphalt and regenerant [20,21,22,23,24]. The above background information fully demonstrates that on the premise of adding regenerants to restore the performance of aged asphalt, the RAP content can be increased. However, there is still a lack of reference standards for the dosage and curing conditions of regenerants.

Asphalt mixtures are road construction materials with viscoelastic properties, and evaluation of their mechanical performance has always been a hot topic in the field of road engineering research [25,26,27]. The dynamic modulus and bending stiffness modulus are necessary parameters for asphalt pavement design [28,29]. Therefore, this study investigated the changes in the mechanical parameters of hot-recycled asphalt mixture under the influence of different factors (regenerant dosage, curing temperature, and curing time) through uniaxial compression dynamic modulus tests and three-point bending tests of small beams. The results of this research can provide a reference for the design of hot-recycled asphalt mixture and contribute to the wider application of RAP in recycled mixture.

## 2. Materials and Methods

### 2.1. Regenerant

The regenerant employed in this study was self-made. The related properties are recorded in Table 1.

### 2.2. RAP

The RAP came from the Changchun–Siping section of the Changyu highway, which had been in service for nearly 15 years. The pavement structure of this section of road consisted of upper, middle, and lower layers. The upper layer was 4 cm modified SMA-16, the middle layer was 5 cm AC-20, and the lower layer was 6 cm AC-25. JTG/T F41-2017 requires that RAP used for recycled mixture has appropriate gradation and strength. Therefore, it was necessary to undertake performance testing to determine whether the milled recycled old material met the corresponding requirements.

#### 2.2.1. Grading of RAP

Normally, the grading design of hot-recycled asphalt mixture is determined based on RAP gradation. However, for the original pavement asphalt mixture, after a long service time, part of the coarse aggregate was refined under the effects of the environment and vehicle load. Therefore, it was necessary to screen the recycled material, determine the gradation of RAP, and then perform the grading design of the hot-recycled asphalt mixture.

**Figure 1:**
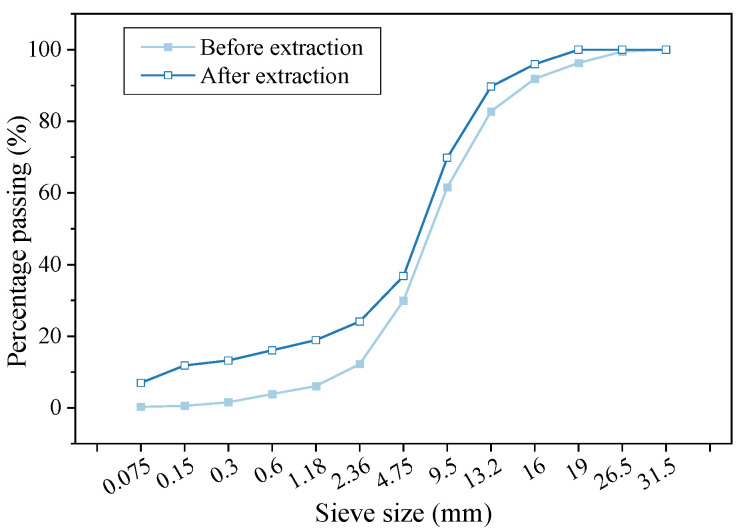
Grading curve of recycled materials before and after extraction.

An asphalt mixture extractor was used to extract the recycled material with trichloroethylene as the solvent. The extracted liquid was distilled using a rotary evaporator for asphalt recovery, and the asphalt content in the RAP was calculated to be 4.2%. The passing rates of each grade aggregate before and after extraction were used to prepare a grading curve, as shown in Figure 1. The linearity of the grading curve before and after extraction was consistent, but the proportion of fine aggregate in the recycled aggregate was higher after extraction. Therefore, the amount of fine aggregate could be considered to increase during the grading design of the hot-recycled asphalt mixture.

#### 2.2.2. Performance Evaluation of RAP

The penetration, 60 °C dynamic viscosity, softening point, and 15 °C ductility of the recycled asphalt were measured in accordance with JTG/T F41-2017, and the results are shown in Table 2. The moisture content, sand equivalent, needle and flake particle content, and crushing value of the RAP aggregate were tested, and the results are shown in Table 3.

### 2.3. Mix Design of Hot-Recycled Asphalt Mixture

**Figure 2:**
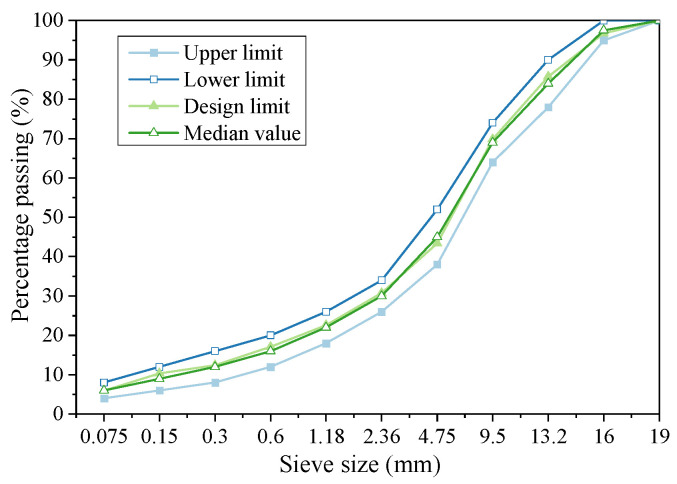
Design grading of hot-recycled asphalt mixture with RAP.

According to the indicators of RAP performance, hot-recycled asphalt mixture of AC-16 is designed to contain 80% RAP, 14% fine aggregates with particle sizes of 0–3 mm, and 6% coarse aggregates with particle sizes greater than 13.2. The grading curve was prepared based on this grading to meet the required upper and lower limits, from JTG F40-2004, as shown in Figure 2.

The optimum asphalt content of the recycled asphalt mixture was determined to be 4.9%. The Marshall results conducted on the basis of JTG E20-2011 are displayed in Table 4.

### 2.4. Experimental Groups

Based on the principle of a single variable, 15 groups of samples were set with the dosage of regenerant, curing time, and curing temperature as shown in Table 5.

### 2.5. Laboratory Experiments

**Figure 3:**
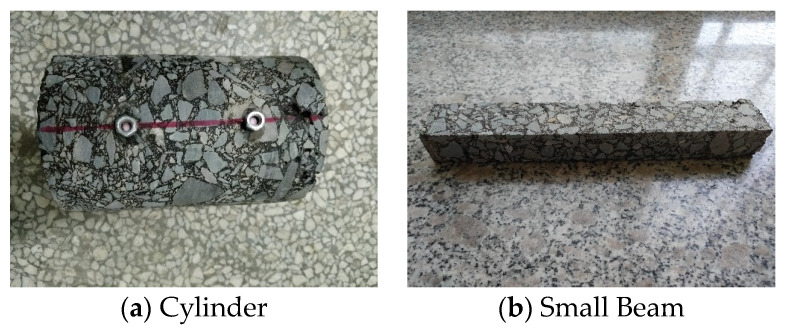
(**a**,**b**) Hot-recycled asphalt mixture specimens.

To eliminate the influence of boundary effects on the experiment, a Superpave gyratory compactor (SGC) was employed to prepare the samples for the dynamic modulus test, and different regenerants were added according to the designed grading. The molded specimens were φ 150 mm × 170 mm (high) cylinders, and then the specimens were cured in the environment described in Table 3. After cooling and forming, a drilling rig was used for coring to obtain a φ 100 mm × 150 mm (high) cylindrical specimen, as shown in Figure 3a. In the three-point bending testing of the small beams, plate-shaped specimens mixed with different regenerants were first formed by rolling according to the designed grading and cured in the environment described in Table 3. After cooling and forming, a cutting machine was used to obtain 250 mm × 30 mm × 35 mm prism beams, as shown in Figure 3b.

#### 2.5.1. Dynamic Modulus

The uniaxial compression dynamic modulus testing of the asphalt mixture was conducted using a universal testing machine (UTM-100) under unconfined conditions according to JTG E20-2011. An offset sine wave axial compressive stress was applied to the specimen at a certain loading frequency, in a selected temperature environment. The recoverable axial strain of the specimen was obtained, the dynamic modulus and phase angle of the asphalt mixture were calculated, and then the mechanical properties of the asphalt mixture were evaluated. The temperatures selected for the experiment were −10 °C, 5 °C, 20 °C, 35 °C, and 50 °C. The loading frequencies of the compressive stress were 0.1 Hz, 0.5 Hz, 1 Hz, 5 Hz, 10 Hz, and 25 Hz.

Based on the time–temperature equivalence principle, the generalized modified sigmoidal model and the Christensen–Anderson–Marasteanu (CAM) model, the master dynamic modulus curve and the phase angle master curve of the hot-recycled asphalt mixture were fitted. The trend and relative position of each master curve were observed, and the effects of the regenerant dosage and curing conditions on the dynamic modulus of the hot-recycled asphalt mixture were summarized.

#### 2.5.2. Three-Point Bending of the Small Beams

At a specific temperature and loading rate, the three-point bending test can be used to determine the maximum load and mid-span deflection of a small beam when it is damaged and then to obtain the bending stiffness modulus of the beam. Based on JTG E20-2011, this test was used to evaluate the low-temperature tensile performance of the asphalt mixture, which is a mechanical parameter used in mixture design. At −20 °C, a small beam prepared from hot-recycled asphalt mixture was loaded at a rate of 50 mm/min by UTM-100 until the small beam was damaged.

## 3. Results and Discussion

### 3.1. Effect of Regenerant Dosage on Modulus

#### 3.1.1. Dynamic Modulus

**Figure 4:**
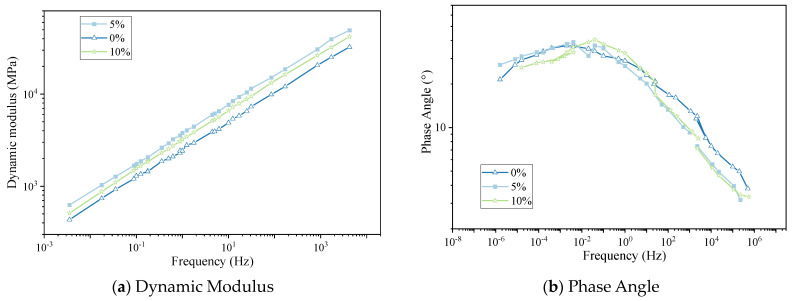
(**a**,**b**) Curves of hot-recycled asphalt mixture with different regenerant dosages.

The master curves of the uniaxial compression dynamic modulus parameters of the hot-recycled asphalt mixture with 0%, 5%, and 10% regenerant dosages are shown in Figure 4.

The following conclusions were obtained by analyzing the relative positions of the curves in the diagram:

(1) The dynamic moduli of hot-recycled asphalt mixture with three dosages of regenerant followed the same trend, and the trend of the values was 0%, 10%, and 5% successively. Thus, increasing the amount of regenerant increased the viscosity of the recycled asphalt, but the dynamic modulus of the hot-recycled asphalt mixture first decreased and then increased with increasing proportions in the mixture.
(2) The regenerant had little effect on the phase angle of the hot-recycled asphalt mixture, and there was some scatter in the curves of the phase angle. However, within the frequency range of the test (0.1–25 Hz), the phase angle of the hot-recycled asphalt mixture was slightly less for 5% than 10% regenerant, and the trend of the dynamic modulus was similar.

#### 3.1.2. Bending Strength Modulus

**Figure 5:**
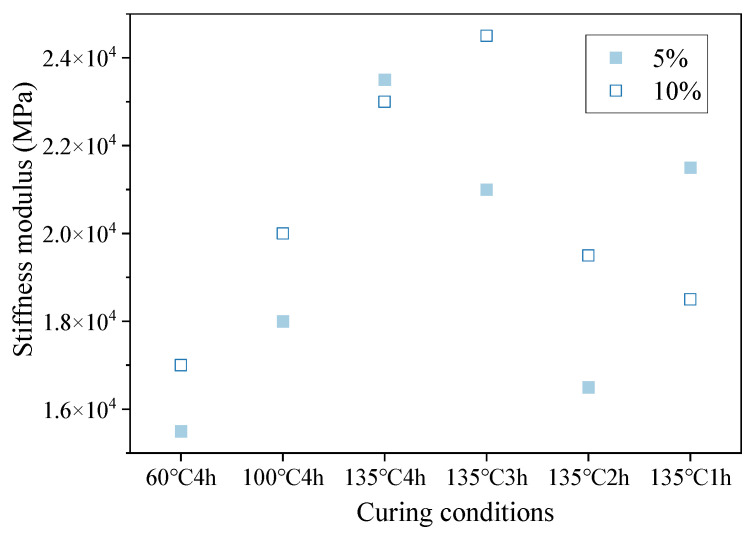
Scatter plot of bending stiffness modulus of hot-recycled asphalt mixture.

In a scatter diagram of data from the three-point bending tests of small beams prepared from hot-recycled asphalt mixture with different dosages of regenerant, the stiffness moduli changed with curing conditions, as shown in Figure 5.

Comparing the relative positions of each point for different curing conditions showed that the bending stiffness modulus followed the same trend for hot-recycled asphalt mixture with 5% and 10% regenerant dosages, and the values of the bending stiffness moduli of the temperature test group were not significantly different. If the minimum values only of the bending stiffness modulus were compared, then the bending stiffness modulus was slightly greater for hot-recycled asphalt mixture with 5% than 10% regenerant dosage.

### 3.2. The Effect of Curing Temperature on Modulus

#### 3.2.1. Dynamic Modulus

**Figure 6:**
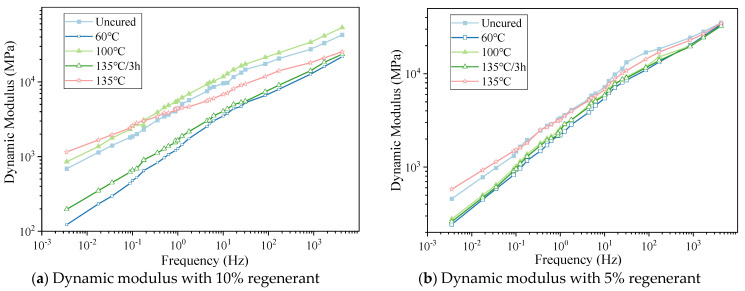
(**a**,**b**) Graphs of the modulus of hot-recycled asphalt mixture under different curing temperatures.

The curves of the uniaxial compression dynamic modulus of the hot-recycled asphalt mixture with regenerant dosages of 10% and 5% and cured at 60 °C, 100 °C, and 135 °C for 4 h are shown in Figure 6a,b, respectively.

After analyzing the relative positions of the primary dynamic modulus curves in the figure, the following conclusions can be drawn:

(1) With a regeneration time of four hours, the dynamic modulus of hot-recycled asphalt mixtures with 5% and 10% rejuvenator dosage was similar under different regeneration temperatures. The dynamic modulus was lower at 60 °C compared with 100 °C. At a regeneration temperature of 135 °C, the dynamic modulus of hot-recycled asphalt mixtures was the highest, approaching the value of asphalt mixture with no added rejuvenator. For hot-recycled asphalt mixtures with 10% rejuvenator dosage, the dynamic modulus at a regeneration temperature of 100 °C was slightly higher than at 135 °C.
(2) When the dosage of the rejuvenator was 5%, the dynamic modulus value of the hot-recycled asphalt mixture at 135 °C for three hours was almost the same as the dynamic modulus value of the hot-recycled asphalt mixture at 100 °C for four hours. When the dosage of the rejuvenator was 10%, the dynamic modulus value of the hot-recycled asphalt mixture at 135 °C for three hours was similar to the dynamic modulus value of the hot-recycled asphalt mixture at 60 °C for four hours. Therefore, when the regeneration temperature varies, adjusting the regeneration time can achieve a similar modulus for the hot-recycled asphalt mixture.
(3) The results of the dynamic modulus under the two rejuvenator dosages indicate that the larger the dosage of the rejuvenator, the more significant was the effect of the regeneration temperature on the dynamic modulus of the hot-recycled asphalt mixture.

#### 3.2.2. Bending Strength Modulus

**Figure 7:**
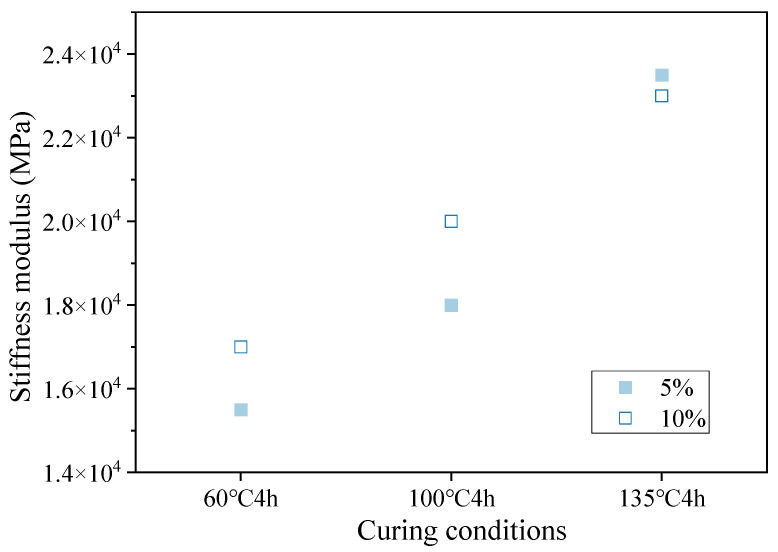
Scatter plot of bending stiffness modulus at different curing temperatures.

The scatter plot of three-point bending test data for small beams in Figure 7 shows the bending stiffness moduli of hot-recycled asphalt mixture with 5% and 10% regenerant at different curing temperatures.

Comparing the relative values of the bending stiffness modulus of the hot-recycled asphalt mixture under different curing temperatures in the figure shows that when the curing time was 4 h, the trends of the bending stiffness modulus for 5% and 8% regenerant were consistent and gradually increased with increasing curing temperature. At the three curing temperatures selected in the experiment, as the curing temperature increased, the bending stiffness modulus of the hot-recycled asphalt mixture continuously increased. That is, within this curing time and temperature range, as the curing temperature increased, the bending stiffness modulus of the hot-recycled asphalt mixture first decreased and then increased.

### 3.3. The Effect of Curing Time on Modulus

#### 3.3.1. Dynamic Modulus

**Figure 8:**
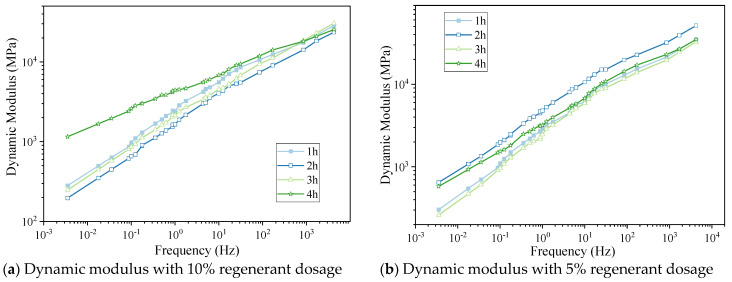
(**a**,**b**) Graphs of the dynamic modulus of the hot-recycled asphalt mixture under different curing times.

Graphs of the uniaxial compression dynamic modulus for hot-recycled asphalt mixture cured at 135 °C for 1 h, 2 h, 3 h, and 4 h for regenerant dosages of 10% and 5% are shown in Figure 8a,b, respectively.

By analyzing the relative positions on each graph, the following conclusions were obtained:

(1) When the curing temperature was 135 °C, the dynamic modulus of the hot-recycled asphalt mixture with a 10% regenerant dosage decreased with curing for 4 h, 1 h, 3 h, and 2 h.
(2) The recovery of the dynamic modulus recovery was generally consistent for hot-recycled asphalt mixture with 5% and 10% regenerant, but the dynamic modulus was higher for specimens cured for 2 h than other lengths of time.
(3) According to the values of the dynamic modulus for two types of regenerant dosages, the larger the regenerant dosage, the more significant was the effect of curing time on the dynamic modulus of the hot-recycled asphalt mixture.

#### 3.3.2. Bending Strength Modulus

**Figure 9:**
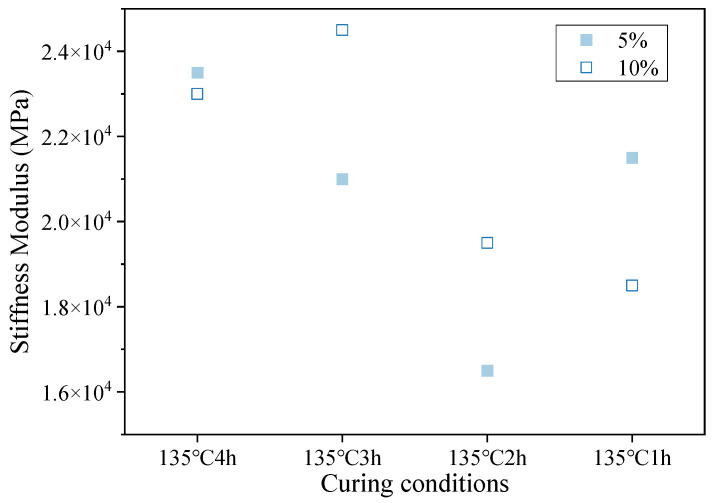
Scatter plot of bending stiffness moduli for different curing times.

According to the three-point bending test data for the small beams, the scatter plot in Figure 9 shows the bending stiffness modulus of the hot-recycled asphalt mixture with 5% and 10% regenerant at different curing temperatures. When the curing temperature was 135 °C, the trends of the bending stiffness modulus for the two levels of recycled agent were basically consistent, and as the curing time increased, the bending stiffness modulus first decreased, then increased, and then decreased again. The bending stiffness modulus of the hot-recycled asphalt mixture cured for 2 h was the smallest, but the bending stiffness modulus of the hot-recycled asphalt mixture cured for 4 h with 10% regenerant dosage was smaller than that cured for 3 h.

### 3.4. Analysis of Factors Influencing the Modulus

#### 3.4.1. Qualitative Analysis of Modulus Changes

**Figure 10:**
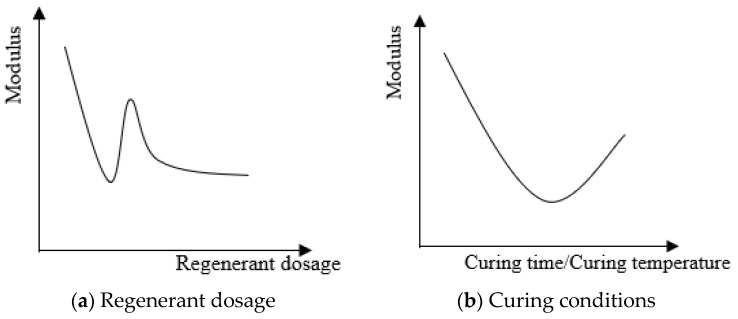
(**a**,**b**) Trends of the modulus of the hot-recycled asphalt mixture.

Overall, with increasing regenerant dosage, the modulus of the hot-recycled asphalt mixture first decreased, then increased, and then decreased. As the curing time or temperature increased, the modulus first decreased and then increased, as shown in Figure 10.

(1) The dynamic modulus and bending stiffness modulus data of the hot-recycled asphalt mixture showed that the dynamic modulus was indeed slightly lower for the 10% than the 5% dosage. Although there was no peak, it satisfied the trend of the modulus curve.
(2) The dynamic modulus of the hot-recycled asphalt mixture with 10% regenerant was slightly larger than that of the control group without the addition of recycled agent, for a curing temperature of 100 °C. However, the dynamic modulus of the hot-recycled asphalt mixture with both types of regenerant dosages reached its minimum at a curing temperature of 60 °C and then increased with increasing curing temperature, which was basically consistent with the overall trend.
(3) Although the dynamic modulus of the hot-recycled asphalt mixture with the 5% regenerant dosage reached its maximum value after 2 h of curing, its overall trend of change followed the basic trend. The dynamic modulus of the hot-recycled asphalt mixture with 10% regenerant first decreased and then increased, with a minimum value after 2 h of curing, which was consistent with the basic trend.
(4) Comparing the minimum bending stiffness modulus of hot-recycled asphalt mixture with different regenerant dosages showed that for the recovery degree of low-temperature performance, the performance was slightly better for hot-recycled asphalt mixture with 5% than 10% regenerant, which was consistent with the changes in basic behavior.
(5) When the curing time was 4 h, as the curing temperature increased, the bending stiffness modulus of the hot-recycled asphalt mixture showed an upwards trend. Although it deviated from the basic trend of first decreasing and then increasing, it also satisfied the latter half of the trend.
(6) When the curing temperature was 135 °C for the hot-recycled asphalt mixture with 10% regenerant, the bending stiffness modulus was lower for the asphalt cured for 4 h than 3 h. However, when the curing time was within 3 h, the bending stiffness modulus of the hot-recycled asphalt mixture with two types of regenerant dosages first decreased and then increased, and the curing temperature was the lowest at 2 h. Therefore, the trend in the bending stiffness modulus within 3 h of curing was consistent with the basic trend, which first decreased and then increased.

#### 3.4.2. Quantitative Analysis of Modulus Changes

To more intuitively observe the changes in the dynamic modulus of the hot-recycled asphalt mixture under different curing conditions, the average change ratio of the dynamic modulus was summarized. This ratio compared the hot-recycled asphalt mixture under different curing conditions to that without curing, as shown in Table 6.

**Figure 11:**
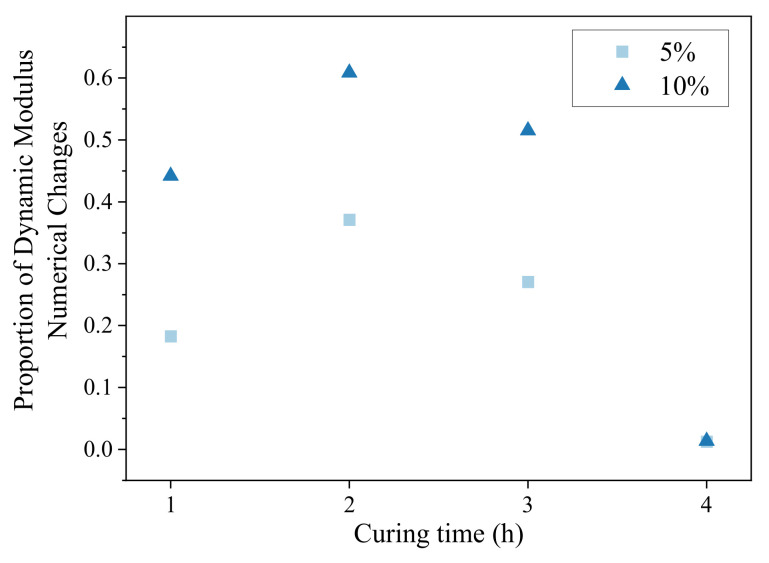
Proportion of numerical changes in dynamic modulus of hot-recycled asphalt mixture after different curing times.

When the curing time was 4 h, the dynamic moduli of the hot-recycled asphalt mixture with two types of regenerant dosages were basically the same as those under the curing environments of 100 °C and 135 °C. Therefore, the dosage of regenerant mainly affected the dynamic modulus of the recycled mixture cured at low temperature. The scatter plot of the change rate of dynamic modulus values with curing time when the curing temperature was 135 °C is shown in Figure 11. The hot-recycled asphalt mixture with a curing time of 4 h was already in short-term ageing, and thus the change rate of the dynamic modulus value for the two types of regenerant dosages was the same. However, when the mixture was not aged, the numerical change-rate curves of the modulus of the mixture with two types of regenerant dosages had a consistent trend and slope.

In Table 7, the average change ratio of the bending stiffness modulus is summarized for the hot-recycled asphalt mixture under different curing conditions and compared with that without curing.

**Figure 12:**
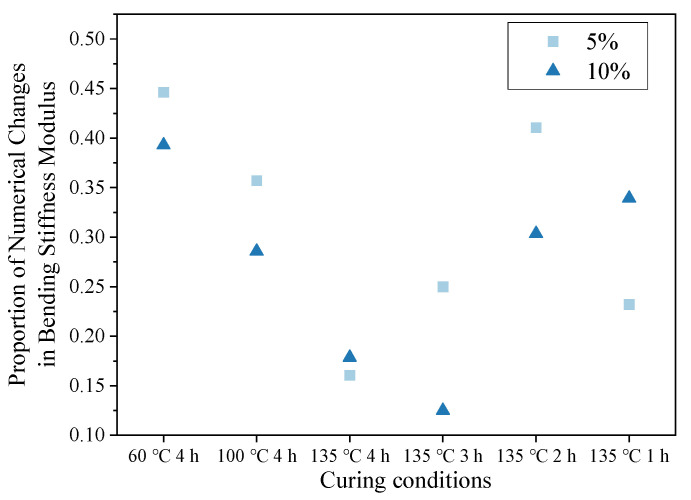
Proportion of numerical changes in bending stiffness modulus under different curing conditions.

Based on the data in the table, a scatter plot of the change rate of the dynamic modulus values with changes in curing conditions is shown in Figure 12. The figure shows that the numerical change-rate curves of the modulus of the mixture with two types of regenerant dosages had a consistent trend and slope.

**Figure 13:**
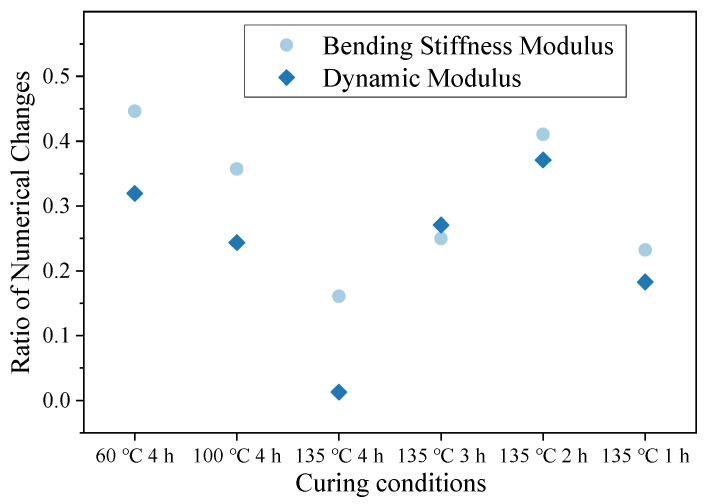
Ratio of numerical changes in dynamic modulus and flexural stiffness modulus of hot-recycled asphalt mixture.

The scatter plot of the change ratio of the dynamic modulus with 5% recycled agent is shown in Figure 13. The trend and slope of the broken line in the figure show that the influence of curing conditions on the dynamic modulus and flexural stiffness modulus of the hot-recycled asphalt mixture was consistent, but the influence on the flexural stiffness modulus was greater.

To further evaluate mechanical parameters of hot-recycled asphalt mixture with RAP, the grey correlation analysis for the correlation degree of influential factors was calculated. This method is always applied to reveal the change among data sequences [30,31,32]. The greater the grey correlation coefficient, the higher is the relevant degree. The dynamic modulus and bending stiffness modulus were taken as the reference sequences, respectively, with the regenerant dosage, curing temperature, and curing time being comparative sequences.

The matrix for influencing factors can be described as follows:

(1) $$\left(\begin{array}{c} X_{1}\\ X_{2}\\ \vdots \\ X_{n} \end{array}\right)=\left(\begin{array}{cccc} x_{1}(1) & x_{1}(2) & \cdots & x_{1}(m)\\ x_{2}(1) & x_{2}(2) & \cdots & x_{2}(m)\\ \vdots & \vdots & & \vdots \\ x_{n}(1) & x_{n}(2) & \cdots & x_{n}(m) \end{array}\right)$$

(2) $$\left(X_{0}\right)=\left(x_{0}(1),x_{0}(2),\cdots ,x_{0}(m)\right)$$

Then, a non-dimension matrix can be derived from the normalization:

(3) $$x_{i}(k)=\frac{X_{i}(k)}{X_{i}(1)}\;\;\;\;\;k=1,\;2,\;\ldots \;m\;\;\;\;\;i=1,\;2,\;\ldots \;m$$

The correlation coefficient can then be calculated:

(4) $$\xi_{i}(k)=\frac{\underset{i}{\min}\;\underset{k}{\min}\left|x_{0}(k)-x_{i}(k)\right|+\rho \;\underset{i}{\max}\;\underset{k}{\max}\left|x_{0}(k)-x_{i}(k)\right|}{\left|x_{0}(k)-x_{i}(k)\right|+\rho \;\underset{i}{\max}\;\underset{k}{\max}\left|x_{0}(k)-x_{i}(k)\right|}$$

where *ρ* is the recognition coefficient, defined as 0.637. Finally, the grey correlation coefficient is calculated as:

(5) $$\varphi_{i}=\frac{1}{N}\sum_{k=1}^{N}\xi_{i}(k)$$

The results of the grey correlation analysis are depicted in Table 8. For the dynamic modulus of the hot-recycled asphalt mixture, the order of correlation degree was curing time > regenerant dosage > curing temperature. The grey correlation coefficient of curing time was 0.7213, followed by the regenerant dosage with a correlation coefficient of 0.6898, indicating the regenerant dosage mattered as well. For bending stiffness modulus, the order of correlation degree was regenerant dosage > curing time > curing temperature. Considering the regenerant dosage and curing time with a close weight of correlation degree, the bending stiffness modulus showed little deviation.

## 4. Conclusions

Based on the uniaxial compression dynamic modulus test and the three-point bending test on the small beams, the results of this study summarize the influence of the regenerant dosage, curing temperature, and curing time on the dynamic modulus and bending stiffness modulus of the hot-recycled asphalt mixture with high RAP.

(1) In the design of hot-recycled asphalt mixture, mixtures with similar responses can be obtained by changing the dosage of regenerant, curing time, curing temperature, and other parameters.
(2) For the dynamic modulus of the hot-recycled asphalt mixture, the influence of the curing time was the largest, followed by the regenerant dosage, and finally the curing temperature. For the bending stiffness modulus, the regenerant dosage had the greatest influence, followed by the curing time, and then the curing temperature.
(3) The influence of each factor was greater for the bending stiffness modulus than the dynamic modulus of the hot-recycled asphalt mixture, and the modulus change rate of the former was approximately 1.5 times that of the latter.

## Figures and Tables

**Table 1:** Technical properties of regenerant.

Testing Items	Indicators	Technical Requirement
Flash point (°C)	228	≥220
Saturates content (%)	14.6	≤30
Viscosity ratio before and after thin-film oven test (%)	1.41	≤3
Mass change after thin-film oven test (%)	1.4	≤4, ≥-4
Density 15 °C (g/cm^3^)	0.935	Measured Value

**Table 2:** Technical Indicators of asphalt in RAP.

Asphalt Type	Penetration (0.1 mm)	Softening Point (°C)	15 °C Ductility (mm)	60 °C Dynamic Viscosity (Pa·s)
Asphalt in RAP	57.1	64.9	14.6	6014.5
90#Base asphalt	80–100	>45	>100	≥140

**Table 3:** Technical Indicators of aggregate in RAP.

Testing Items	Indicators	Technical Requirement
Moisture content (%)	0.17	≤3
Maximum particle size (mm)	26.50	≤26.5
Density (g/m^3^)	2.74	≥2.45
Crushing value (%)	17.30	≤30
Needle and flake particle content (%)	8.90	≤15

**Table 4:** Marshall Results of the Hot-Recycled Asphalt Mixture.

Bulk-Specific Gravity (g/cm^3^)	Marshall Stability (kN)	Air Voids (%)	Flow Value (0.1 mm)	VMA (%)	VFA (%)
2.472	13.59	3.53	34.10	13.45	73.75

**Table 5:** Groups of Samples for the Hot-Recycled Asphalt Mixture Test.

Number	Dosage of Regenerant (%)	Curing Condition	Number	Dosage of Regenerant (%)	Curing Condition
1-1	10	Room temperature storage	2-1	5	Room temperature storage
1-2	10	60 °C, 4 h	2-2	5	60 °C, 4 h
1-3	10	100 °C, 4 h	2-3	5	100 °C, 4 h
1-4	10	135 °C, 1 h	2-4	5	135 °C, 1 h
1-5	10	135 °C, 2 h	2-5	5	135 °C, 2 h
1-6	10	135 °C, 3 h	2-6	5	135 °C, 3 h
1-7	10	135 °C, 4 h	2-7	5	135 °C, 4 h

**Table 6:** The Proportion of Dynamic Modulus Change of Hot-Recycled Asphalt Mixture under Different Curing Conditions.

	Curing Condition	60 °C 4 h	100 °C 4 h	135 °C 4 h	135 °C 3 h	135 °C 2 h	135 °C 1 h
Regenerant Dosage							
5%		0.31940	0.24357	0.01269	0.27069	0.37109	0.18280
10%		0.68369	0.24881	0.01346	0.51502	0.60846	0.44191

**Table 7:** Proportion of Changes in the Bending Stiffness Modulus of Hot-Recycled Asphalt Mixture under Different Curing Conditions.

	Curing Condition	60 °C 4 h	100 °C 4 h	135 °C 4 h	135 °C 3 h	135 °C 2 h	135 °C 1 h
Regenerant Dosage							
5%		0.44643	0.35714	0.16071	0.25029	0.41071	0.23214
10%		0.39286	0.28571	0.17857	0.12586	0.30357	0.33929

**Table 8:** Results of Grey Correlation Coefficients.

Modulus	Grey Correlation Coefficient		
	Regenerant Dosage	Curing Temperature	Curing Time
Dynamic modulus	0.6898	0.5747	0.7213
Bending stiffness modulus	0.6767	0.5927	0.6525

## Data Availability

Not applicable.
